# Chronic Sigma 1 receptor activation alleviates right ventricular dysfunction secondary to pulmonary arterial hypertension

**DOI:** 10.1080/21655979.2022.2065953

**Published:** 2022-04-27

**Authors:** Yazhou Sun, Weiguo Wan, Xin Zhao, Xueyu Han, Tianxin Ye, Xiaoli Chen, Qian Ran, Xiukun Wang, Xin Liu, Chuan Qu, Shaobo Shi, Cui Zhang, Bo Yang

**Affiliations:** a Department of Cardiology, Renmin Hospital of Wuhan University, Wuhan, Hubei, China; b Cardiovascular Research Institute, Wuhan University, Wuhan, Hubei, China; c Hubei Key Laboratory of Cardiology, Wuhan, Hubei, China

**Keywords:** Sigma 1 receptor, right ventricular dysfunction, pulmonary arterial hypertension, oxidative stress

## Abstract

Sigma 1 receptor (S1R) has shown a preferable protective effect on left ventricular function, but whether it protects right ventricular (RV) function is still elusive.This study aimed to determine the effects of S1R on RV dysfunction secondary to pulmonary arterial hypertension.Sixty wild-type male Sprague–Dawley rats were randomly divided into the control group, the fluvoxamine group, the pulmonary arterial hypertension group and the pulmonary arterial hypertension combined with fluvoxamine group. Monocrotaline (60 mg/kg) was administered to induce pulmonary arterial hypertension, and fluvoxamine was given for 21 consecutive days to activate S1R after one week of monocrotaline administration. Echocardiographic, serologic, and histologic parameters, qRT-PCR, and western blotting were conducted after 4 weeks of monocrotaline administration.The expression of S1R was decreased in the right ventricle in pulmonary arterial hypertension. TAPSE, and the FAC of the right ventricle were significantly decreased, and RV EDP and the plasma concentration of N-terminal pro-B-type natriuretic peptide was increased in the pulmonary arterial hypertension group, but fluvoxamine partly restored those abnormalities (all P < 0.05). Moreover, pulmonary arteriole remodeling, and fibrosis and hypertrophy in the RV were shown in the pulmonary arterial hypertension group; interestingly, fluvoxamine recovered RV structural remodeling (all P < 0.05) but neither alleviated pulmonary arteriole remodeling nor reduced pulmonary artery pressure. Furthermore, S1R activation protects RV function by upgrading the NRF 2/HO 1-mediated antioxidant stress pathway. In conclusion, chronic S1R activation ameliorates structural remodeling and RV dysfunction secondary to pulmonary arterial hypertension without altering pulmonary artery pressure.

## Highlights

- S1R expression was reduced in right ventricle with right ventricular dysfunction.
- Activation of S1R directly improved right ventricular dysfunction.
- Activation of S1R directly alleviated right ventricular remodeling.
- Activation of S1R reduced oxidative stress in right ventricular dysfunction.

## 1. Introduction

Pulmonary arterial hypertension (PAH) is a chronic disease characterized by a progressive elevation in mean pulmonary artery pressure (mPAP≥ 25 mmHg) associated with pulmonary artery inflammation, abnormal remodeling, and increased right ventricular (RV) afterload, which leads to right heart failure and death. [1,2] Although significant advances have been made in pharmacological strategies in recent years, including calcium channel antagonists, endothelin receptor antagonists, phosphodiesterase type 5 inhibitors, and prostacyclin analogs, etc., the morbidity and mortality in patients with PAH remain high, with a 5-year survival rate of only 50%. [1,3] New treatment strategies are necessary for improving the prognosis of patients with PAH.

Oxidative stress is recognized to increase in the lungs of the experimental rodent PAH model and it contributes to their PAH development. [4,5] Furthermore, elevated oxidative stress is found in the lungs of PAH patients compared with healthy controls.[6] Oxidative stress has been suggested to play a pivotal role in pulmonary vascular remodeling, which in turn increases the right ventricle (RV) afterload, leading to RV hypertrophy and ultimately RV failure.[5] However, the precise pathogenic mechanism underlying RV injury and remodeling remains unclear.

Numerous studies have demonstrated that the stress-responsive transcription factor nuclear factor-erythroid 2 p45-related factor 2 (NRF 2) regulates many cytoprotective enzymes to maintain the stable states of reactive oxygen species (ROS) under physiological conditions. [7–9] The activity of NRF 2 was increased in the oxidative stress response to mitigate the damage of excessive ROS. [10,11] In addition, pharmacological activation of NRF 2 has been shown to alleviate fibrosis and inflammation of the RV in an experimental PAH model. [12,13]

Recently, emerging evidence has shown that the brain Sigma 1 receptor (S1R) contributes to relieving heart hypertrophy [14–16], ischemia‑reperfusion injury,[17] atrial fibrillation[18], and heart failure. [19,20] Similarly, previous studies have revealed key roles of S1R in reducing oxidative stress in a variety of cells, including neurons, retinal Müller glial cells and tissues such as the liver and lung. [21–24] In particular, decreased NRF 2 activation was also an important condition of oxidative stress injury caused by dysregulation of S1R.[21] Furthermore, modulation of NRF 2 activation is critical for oxidative stress protection mediated by S1R activation in retinal cone photoreceptor cells.[25] To the best of our knowledge, less attention has been given to the direct role of S1R in the RV, especially in right ventricle failure.

Therefore, we speculated that pharmacological activation of S1R might alleviate RV remodeling and attenuate RV dysfunction in a rat PAH model by diminishing oxidative stress associated with the regulation of NRF 2 activity.

## 2. Materials and methods

### 2.1 Animals

Sixty male Sprague–Dawley rats weighing 200 g to 220 g were purchased from Hunan SJA Laboratory Animal Co., Ltd. (Changsha; Hunan Provence; China) and were kept in the animal experimental administration of Renmin Hospital of Wuhan University. Animals were kept in specific pathogen-free grade environments and could freely obtain food and water. Isoflurane (2%) was used for rat anesthesia. The experiments were approved by Wuhan University (SY 2019015, the ethics committee of Wuhan third hospital-tongren hospital of Wuhan university).

The animals were randomly divided into four groups (n = 15 each group) as follows: the control group (CTL), the fluvoxamine group (Flu), the pulmonary arterial hypertension group (PAH), and the PAH+fluvoxamine group (P + F). Rodents in CTL and Flu were injected intraperitoneally (IP) with 0.9% sterile normal saline (NS) after one week of adaptation, while animals in PAH and P + F were injected IP with monocrotaline (60 mg/kg; MedChemExpress; HY-N0750) [26–28] to induce PAH. The pharmacological intervention was performed in the second week after the MCT injection. Animals in CTL and PAH were injected IP with NS daily over the following 21 days; however, rodents in Flu and P + F were administered fluvoxamine (12 mg/kg; Aladdin, F129671; Shanghai, China), which has high S1R affinity[14], from the second week to the last week for 21 days in total. Animals were euthanized for further studies in the fourth week post-MCT injection.

### 2.2 Echocardiography

Transthoracic echocardiography was performed according to a previously published method on the fourth week after MCT injection. [26,29,30] Briefly, rodents were anesthetized with isoflurane (2%). Echocardiography was performed using a high-resolution in vivo imaging system (VINNO 6 VET Ultrasound System, Suzhou, Jiangsu Provence; China) equipped with an 18 MHz probe. M-mode ultrasound examination was performed after the obtained 2D image was stabilized. The systolic function of the LV is represented as left ventricular ejection fraction (LVEF) = LV EDV-LV ESV)/LV EDV x 100%), and EDV and ESV represent the end-diastolic volume and end-systolic volume of the LV, respectively. Pulse-wave pulmonary outflow Doppler was recorded in the parasternal view at the level of the aortic valve. In the apical 4-chamber view, we measured the tricuspid annular plane systolic excursion (TAPSE), RV end-systolic area (RVSA), and RV end-diastolic area (RVDA). Systolic function of RV is represented as fraction of area change (FAC) = (RVDA- RVSA)/RVDA×100%.

### 2.3 Hemodynamics

The intrusive hemodynamic test was performed as described previously[31], animals were anesthetized and then placed on the electric blanket. The right carotid vein was careful bluntly separated and isolated. Then, a PU tube connected to a multichannel physiological monitoring system (Biopac MP150) was meticulously implanted into the RV and pulmonary artery. Mean pulmonary arterial pressure(mPAP), right ventricular end-systolic pressure (RV ESP), and right ventricular end diastolic pressure (RV EDP) were measured with LabChart 8 software (AD Instruments, Dunedin, New Zealand).

### 2.4 Serological analysis

A rat N-terminal pro-B-type natriuretic peptide (NT-pro BNP) ELISA Kit (ELK Biotechnology; ELK8211; Wuhan, China) was used to detect the plasma level of NT-pro BNP according to the ELISA kit instructions.

Biochemical examinations were performed to probe the alterations of serum SOD and malondialdehyde (MDA) in serum, which indicated oxidant defense. The concentration was measured by using corresponding biochemical assay kits according to the manufacturer’s instructions (ELK Biotechnology; A001-3 and A003-1, Wuhan, China).

### 2.5 Histological analysis

Rats were euthanized after transthoracic echocardiography, and then the heart and lung were rapidly removed for further analysis. RV hypertrophy was measured by calculating the ratio of the RV to left ventricle (LV) plus septum (S) (RV/LV + S)[32]. The RV tissues and the lung tissues were removed immediately and fixed in a 10% formalin solution. The tissues were sliced and dyed according to standard histological methods, including Masson’s trichrome staining, wheat germ agglutinin (WGA) staining, and hematoxylin-eosin (HE) staining. Masson’s trichrome staining and WGA staining were applied to the RV while HE staining were applied to the lung. The extent of fibrosis (blue–green fibrosis) and the cross-section of cardiomyocytes were quantified using ImageJ software (National Institutes of Health, Bethesda, MD, USA). Pulmonary vascular remodeling was evaluated by determining the wall thickness percentage (WT%) = [(external diameter − internal diameter)/external diameter×100 and wall area percentage (WA%) = [(total area − luminal area)/total area] ×100 of 10 arterioles per rat.[33] All analyses were performed in a blinded manner.

### 2.6 ROS generation analysis

ROS generation in the RV tissues was measured by dihydroethidium (DHE) fluorescence staining according to a standard procedure. The fluorescence intensity was measured by ImageJ software.

### 2.7 Immunofluorescence staining

To determine whether S1R is expressed in the pulmonary arterioles, sections of lung tissues were permeabilized with 0.01% Triton X-100 and blocked with 5% normal bovine serum for 1 h at room temperature. Then, the sections were fixed and stained using antibodies against S1R and α-SMA (α-smooth muscle actin) at 4°C overnight, followed by tetramethylrhodamine isothiocyanate (TRITC) coupled with goat anti-rabbit antibody (1:100), which was stained at room temperature in darkness for 1 h. DAPI staining was performed for 8 min. Images were obtained by microscopy (ScanScope CS, Aperio, Olympus).

### 2.8 Western blotting

Rats were anesthetized in a deep anesthetic state. Then, the RV were removed and rapidly frozen in liquid nitrogen. Protein extraction and western blotting were performed using standard techniques. The membranes were probed with antibodies against S1R (1:1000, Abcam, ab253192), NRF 2 (1:1000, Proteintech, 16,396-1-AP), NADPH oxidase 2 (NOX2, 1:1000, Proteintech, 19,013-1-AP), NADPH oxidase 4 (NOX4, 1:1000, Abcam, ab133303), heme oxygenase-1 (HO 1, 1:3000, Abcam, ab68477), and collagen I (1:500, Abcam, ab260043). The levels of diverse proteins were normalized to glyceraldehyde-3-phosphate dehydrogenase (GAPDH, 1:10,000, Abcam, ab181602) levels for further analysis. The images of the western blots were scanned and analyzed using ImageJ software.

### 2.9 qRT-PCR

Quantitative real-time polymerase chain reaction (qRT-PCR) analysis was performed to determine the relative expression of NRF 2 and HO 1 using gene-specific primers as described previously. Briefly, total RNA in the right ventricular tissues was extracted using a RNeasy kit (ELK Biotechnology; EP013, Wuhan, China). The primers were designed by the Primer Express software package. The level of diverse RNA was normalized to GAPDH. The primer sequences of NRF 2, HO 1 and GAPDH were shown in Table 1. ABI Prism 7900 sequence detection system software (version 2.2) was used to analyze the data.

**Table:** 

	CTL	Flu	P value vs.CTL	PAH	P value vs.CTL	P+F	P value vs.PAH
RV EDP (mmHg)	4.34±1.29	2.45±1.25	0.32	9.60±2.09	<0.001	5.90±1.94	0.02
RV ESP (mmHg)	22.82±2.31	24.12±8.20	0.99	62.71±22.93	0.009	54.89±23.06	0.88
TAPSE (mm)	3.33±0.09	3.23±0.07	0.30	2.17±0.22	<0.001	2.82±0.20	<0.001
FAC(%)	50.53±2.05	48.68±5.62	0.53	30.24±6.75	<0.001	41.35±4.17	<0.001
LVEF(%)	81.98±2.64	80.74±4.52	0.58	82.06±3.57	0.97	80.60±4.23	0.51
NT-pro BNP(pg/ml)	0.51±0.10	0.53±0.12	0.99	1.63±0.17	<0.001	0.80±0.23	<0.001
mPAP(mmHg)	17.68±3.94	19.01±2.94	0.99	43.22±11.78	0.002	43.29±13.25	>0.99
WA%(%)	35.66±6.22	36.52±6.21	0.53	86.39±4.06	<0.001	84.55±4.24	0.19
WT%(%)	21.26±2.99	21.03±6.34	0.89	64.98±7.15	<0.001	64.83±7.63	0.92
Fibrosis in perivascular space of RV(%)	2.38±0.63	2.28±0.05	0.87	11.04±0.23	<0.001	3.18±0.51	<0.001
Fibrosis in interstitial space of RV(%)	1.08±0.05	1.13±0.17	0.93	7.23±1.26	<0.001	2.06±0.19	<0.001
Mean cross-sectional area(μm^2^)	244.36±10.14	246.13±9.68	0.95	560.25±18.84	<0.001	416.32±28.38	<0.001
RV/(LV+S)	0.32±0.02	0.34±0.02	0.98	0.76±0.04	<0.001	0.58±0.01	<0.001
OD value	49.31±1.34	57.09±5.63	0.10	74.43±3.51	<0.001	52.70±4.54	<0.001
MDA (nmol/ml)	9.09±2.90	8.83±3.16	0.99	27.30±6.70	<0.001	15.77±2.65	<0.001
SOD(U/ml)	326.27±58.61	329.07±57.11	0.99	66.34±19.04	<0.001	210.75±30.16	<0.001
S1R/GAPDH	0.72±0.03	0.73±0.04	0.81	0.09±0.01	<0.001	0.35±0.01	<0.001
NOX 2/GAPDH	0.13±0.03	0.14±0.05	0.96	0.57±0.08	0.004	0.51±0.12	0.61
NOX 4/GAPDH	0.16±0.04	0.15±0.05	0.94	0.79±0.10	0.005	0.69±0.19	0.56
NRF 2/GAPDH	0.60±0.09	0.61±0.07	0.96	0.09±0.02	<0.001	0.31±0.04	0.03
HO 1/GAPDH	0.64±0.03	0.63±0.03	0.81	0.086±0.02	<0.001	0.22±0.02	0.005
Collagen I/GAPDH	0.14±0.02	0.14±0.03	0.95	0.53±0.07	<0.001	0.31±0.02	0.006
Fold change in expression of NRF 2	0.99±0.018	0.91±0.02	0.002	0.44±0.03	<0.001	0.72±0.02	<0.001
Fold change in expression of HO 1	1.08±0.11	1.00±0.10	0.22	0.46±0.03	<0.001	0.69±0.03	0.006

**Table 1.** RT-PCT primers

Gene	Primer	Sequence (5’-3’)	Size(bp)
R-GAPDH	Sense	AACAGCAACTCCCATTCTTCC	164
	Antisense	TGGTCCAGGGTTTCTTACTCC	
R-Nrf2	Sense	CAACTGGATGAAGAGACCGGAG	294
	Antisense	TATGCTGCTTAAATCAGTCATGGC	
R-HO-1	Sense	GTGACAGAAGAGGCTAAGACCG	290
	Antisense	GGCCAACACTGCATTTACATGG	

### 2.10 Statistical analysis

All statistical analyses were performed by IBM SPSS 23 or GraphPad Prism 8. Continuous variables are expressed as the means ± standard deviation, and proportions are reported as percentages. Comparisons between groups were performed with analysis of variance (ANOVA). Statistical significance was defined as P ≤ 0.05.

## 3. Results

In the present study, we focused more on the role of S1R receptors in right ventricular dysfunction secondary to pulmonary hypertension and its downstream mechanisms. Activation of S1R improved right ventricular function but had no significant effect on pulmonary artery pressure by ultrasound and invasive hemodynamic assays. Oxidative stress is a potential downstream mechanism of S1R activation, and NRF2/HO1 may be the main mechanism by which S1R acts.

### 3.1 S1R activation alleviates the RV dysfunction

**Figure 1.**
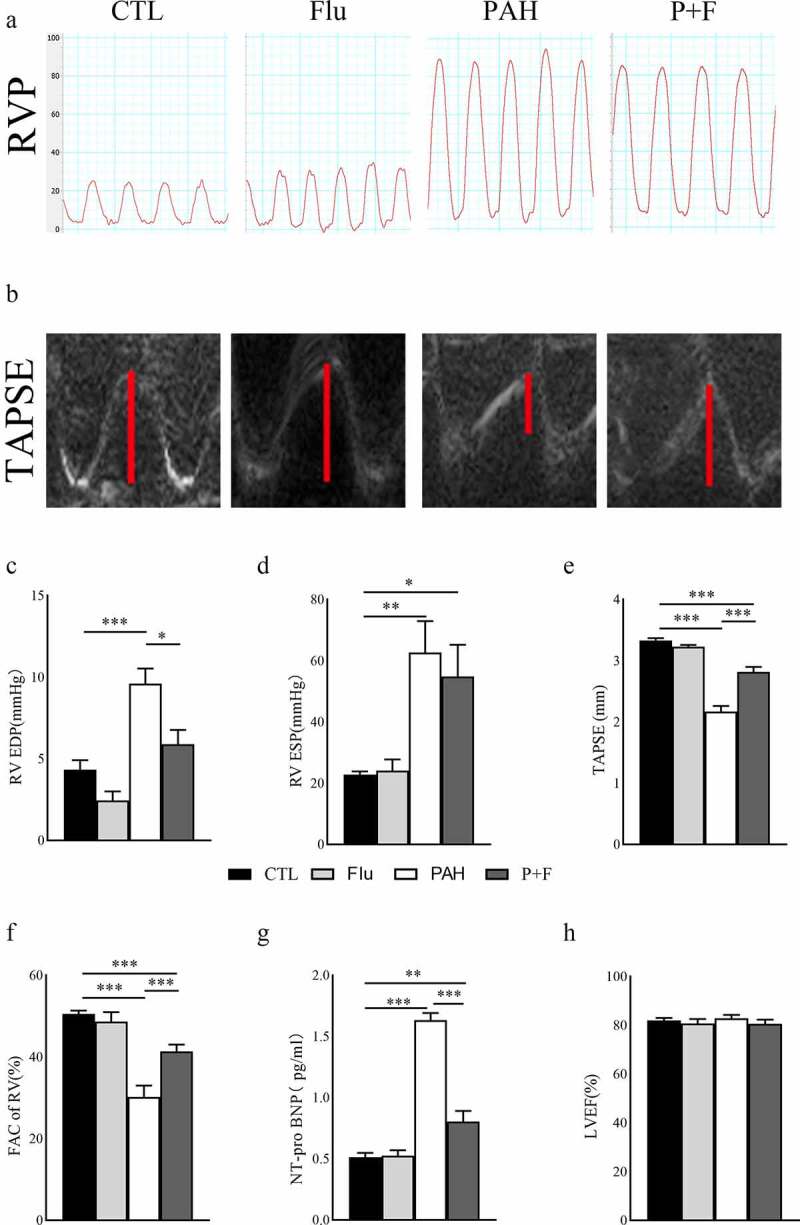
The RV function variation in PAH animals and fluvoxamine-treated PAH rats. (a) The RVP in the four groups, (b) The TAPSE in the four groups; (c) The statistical results of RV EDP(n = 5), (d) the statistical results of RV ESP(n = 5), (e) The statistical results of TAPSE (n = 6), (f) The statistical results of RV FAC (n = 6), (g) Quantification of the plasma concentration of NT pro-BNP (n = 8), (h) The LVEF in the four groups (n = 6), RVP right ventricular pressure, TAPSE tricuspid annular plane systolic excursion, RV EDP right ventricular end-diastolic pressure, RV ESP right ventricular end-systolic pressure, FAC fraction of area change, NT-pro BNP N-terminal pro-B-type natriuretic peptide, LVEF left ventricular ejection fraction, * P < 0.05, ** P < 0.01, *** P < 0.001.

At week 4 post-MCT injection, we proceeded with transthoracic echocardiography and intrusive hemodynamic test to detect the heart function variation of animals. PAH with RV dysfunction was evident in the PAH group vs. CTL group on echocardiography and hemodynamics, demonstrated both by a shortened TAPSE (Figure 1b,1e), elevated RV ESP (Figure 1a,1d) and increased RV EDP (Figure 1a,1c). More directly, RV function was impaired, as evidenced by the decreased FAC in the PAH group compared to the CTL group (figure 1f). As expected, fluvoxamine administration alone did not alter RV function, as demonstrated by the indicators mentioned above, and it was not different in Flu vs. CTL animals (Figure 1a-1f). Compared with the PAH group, RV function was improved in the P + F group, evident as an increase in TAPSE (Figure 1b,1e) and decrease in RV EDP (Figure 1a,1c). Similarly, the FAC of the RV was partly restored in the P + F group (figure 1f), However, the RV ESP in the P + F group was consistent with the PAH group (Figure 1a,1d).

In addition, the plasma level of NT-pro BNP (Figure 1g) was significantly elevated in the PAH group compared with the CTL group, indicating worsening heart function in the PAH rat model. The heightened concentration of NT-pro BNP in PAH was reversed by fluvoxamine, as shown in the P + F group vs. the PAH group (Figure 1g), further underlying the improvement of RV systolic function. LV systolic function was not altered by MCT administration, as verified by the constant LVEF in the four experimental groups (Figure 1h).

### 3.2 S1R activation does not mitigate pulmonary artery pressure and the structural remodeling of pulmonary arteriole

**Figure 2.**
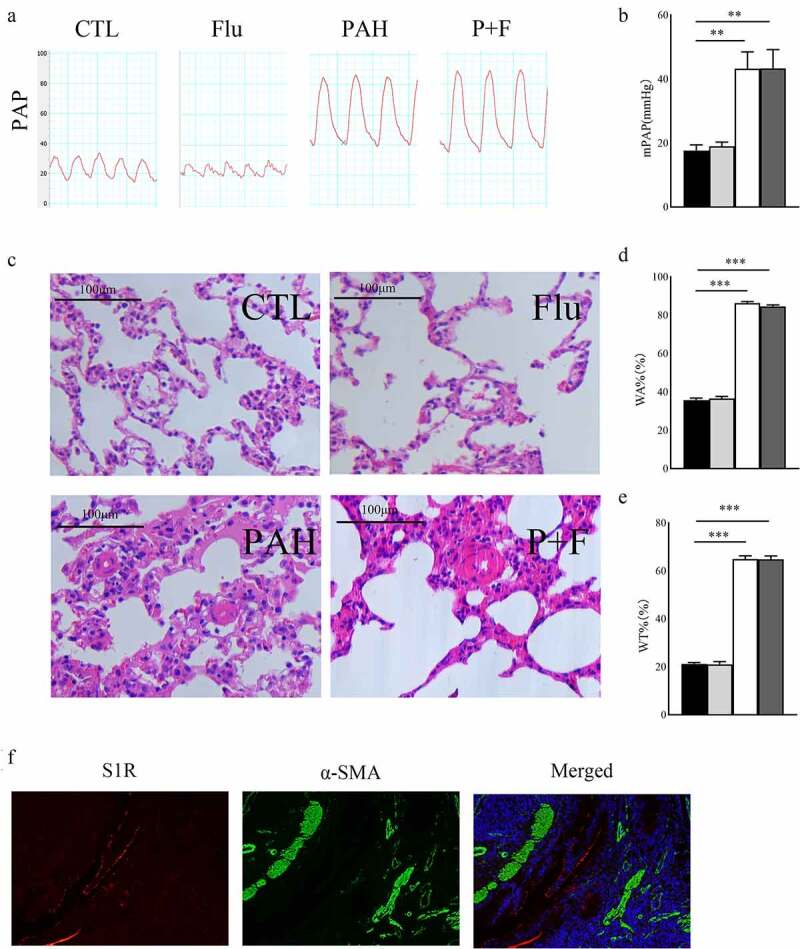
Structural remodeling in the pulmonary arteriole. (a) the PAP in the four groups, (b) The statistical results of mPAP(n = 5), (c) HE staining of the lung tissue (original magnification × 400), (d) The WA% in the four groups (n = 30 pulmonary arterioles from 3 rats) (e) The WT% (n = 30 pulmonary arterioles from 3 rats), (f). the expression status of S1R in pulmonary arterioles, S1R (red), α-SMA (green), DAPI (blue). HE hematoxylin-eosin staining, WT%, wall thickness percentage, WA% wall area percentage, PAP pulmonary artery pressure, mPAP mean pulmonary artery pressure, α-SMA α-smooth muscle actin * P < 0.05, ** P < 0.01, *** P < 0.001.

The pulmonary arterial hypertension model was established by significantly elevated mPAP in the PAH group (Figure 2a, 2b). Chronic S1R activation did not affect pulmonary artery pressure in normal rats (Figure 2a, 2b), nor did it decrease pulmonary artery pressure in PAH rats (Figure 2a, 2b).

Pulmonary arteriole remodeling was the most notable characteristic of PAH induced by MCT. Pulmonary arterial wall thickening was observed in PAH rats, as shown by the increased WA% in the PAH group compared with the CTL group (Figure 2c, 2d) as well as the WT% (Figure 2c, 2e). However, fluvoxamine significantly deceased WT% in the PAH group (Figure 2c, 2d, 2e), indicating that pulmonary arteriole structural remodeling was not relieved by S1R activation. Activation of S1R did not affect pulmonary microvascular remodeling and pulmonary artery pressure was associated with the absence of S1R expression in pulmonary arterioles (figure 2f).

### 3.3 S1R activation relieves the structural remodeling of RV

**Figure 3.**
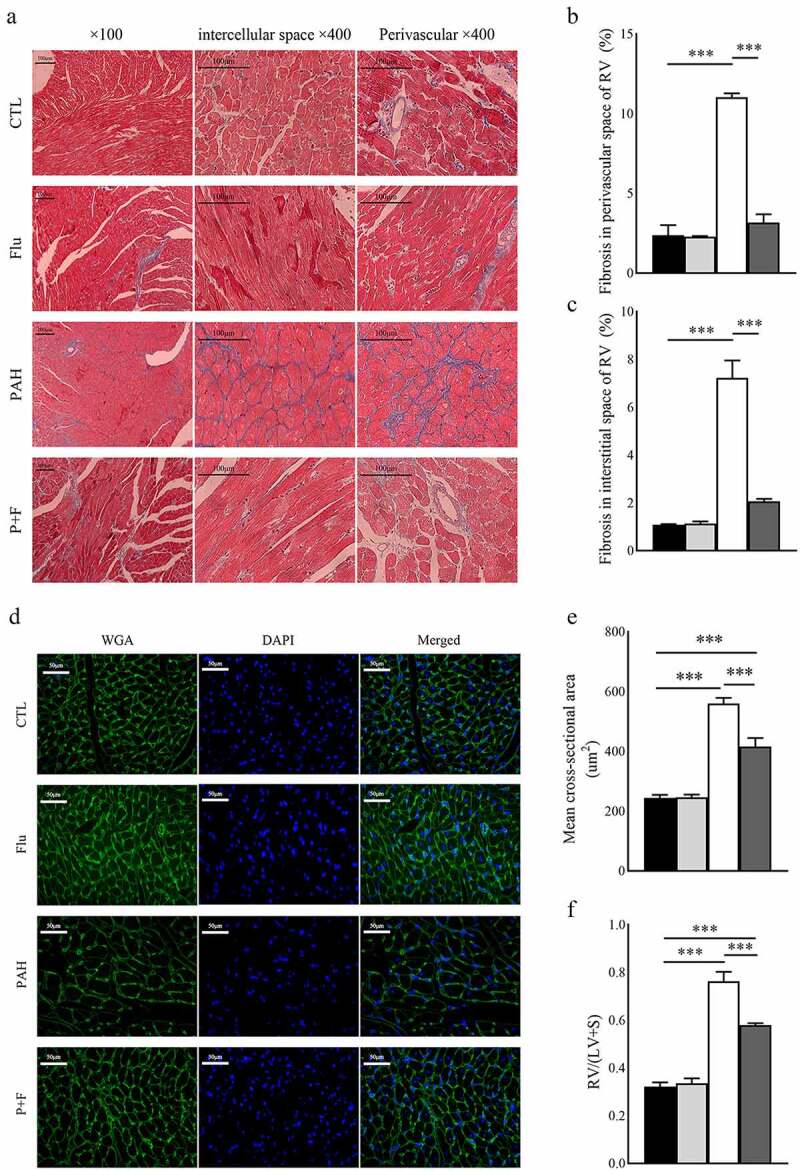
Structural remodeling in the RV. (a) Representative images of Masson’s staining in the RV (original magnification ×100 and ×400), (b) quantification of fibrosis in the perivascular space (n = 3), (c) quantification of fibrosis in the interstitial space (n = 3), (d) representative image of wheat germ agglutinin (WGA) staining in the RV (original magnification× 400) (n = 3), (e) quantification of the mean cross-sectional area of cardiomyocytes in the RV, (f) the quantification of RV/(LV+S) (n = 6), *** P < 0.001.

Increased collagen deposition in the RV of PAH rats was evident, as shown by the significantly extended area of fibrosis in Masson’s trichrome staining both around blood vessels (Figure 3a,3b) and in the interstitial space (Figure 3a,3c). In contrast, fluvoxamine did not change the fibrosis of the RV in normal animals around blood vessels (Figure 3a,3b, 3c). Notably, fibrosis in the RV of PAH rats was markedly decreased by S1R activation by fluvoxamine (Figure 3a,3b, 3c).

MCT significantly increased the absolute RV/(LV+S) (figure 3f), validating the severe RV hypertrophy of PAH. Comparably, at the microlevel, the same results were observed, as shown in the WGA staining test (Figure 3d,3e). The rats in the P + F group had a significant reduction in the RV/(LV + S) (figure 3f) as well as in the average cross-sectional area of cardiomyocytes exhibited by WGA staining (Figure 3d,3e).

### 3.4 S1R activation weakens the oxidative stress induced by MCT

**Figure 4.**
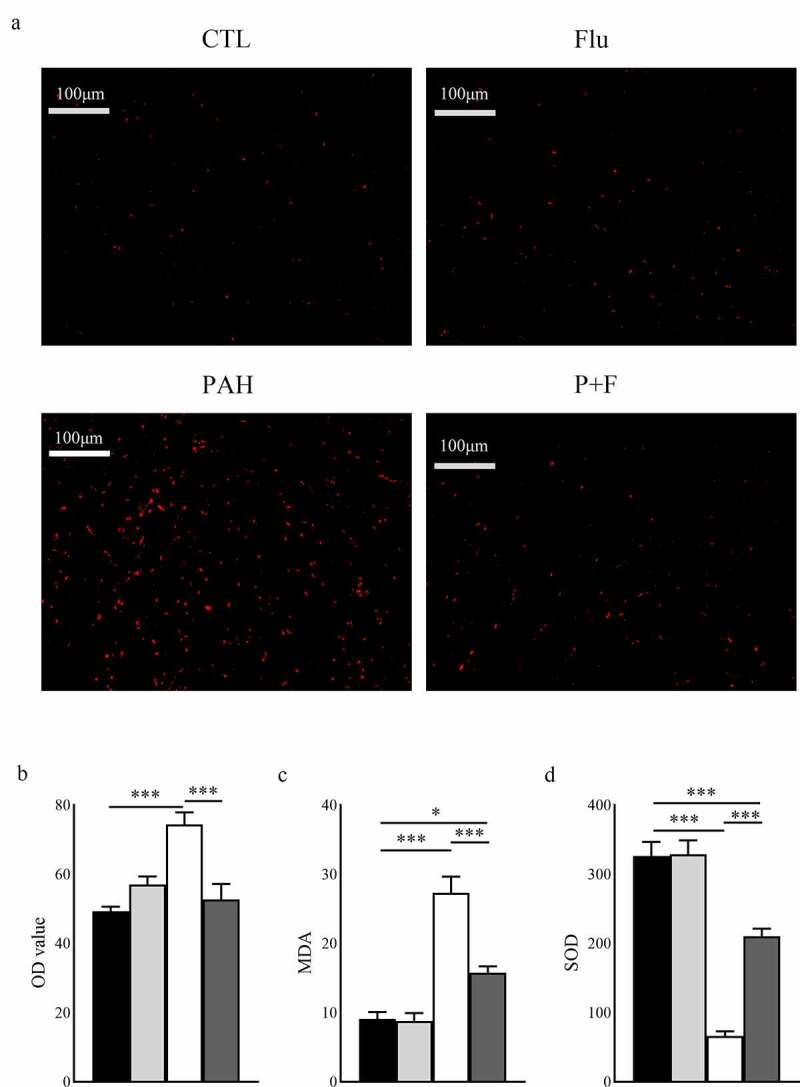
Oxidative stress in the RV and serum. (a) Representative dihydroethidium (DHE) fluorescence staining in the RV. (b) Quantification of ROS in the RV (n = 3). (c) Quantification of the plasma concentration of MDA (n = 8). (d) Quantification of the plasma concentration of SOD (n = 8). ROS reactive oxygen species, original magnification ×200, * P < 0.05, *** P < 0.001.

Measured by DHE staining, the content of reactive oxygen species in RV was symbolically increased in the PAH group compared with their counterparts in the CTL group (Figure 4a,4b). Moreover, the plasma concentration of MDA, suggestive of lipid peroxidation, was also increased (Figure 4c), and the antioxidant enzyme SOD in the serum was downregulated (Figure 4d). Interestingly, this soaring oxidative stress level was partially alleviated by fluvoxamine administration, as demonstrated by DHE (Figure 4a,4b) and the serum concentration of MDA (Figure 4c), and the antioxidant abilities were restored (Figure 4d), although it did not affect the normal animals.

### 3.5 The underlying mechanisms by which S1R activation resulted in the morphological and functional phenomena

**Figure 5.**
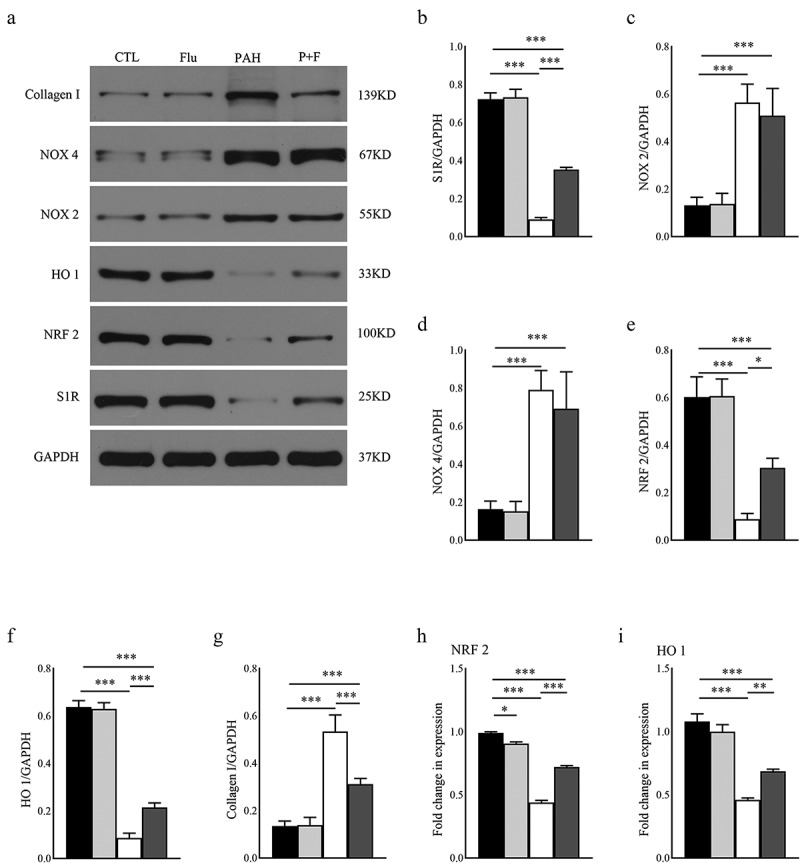
Western blot assay and qRT-PCR of the RV tissues. (a) Blot images of S1R, NOX 2, NOX 4, NRF 2, HO 1, and collagen I, (b-h). Protein levels of S1R, NOX 2, NOX 4, NRF 2, HO 1, and Collagen I normalized to GAPDH (n = 3); (i) RNA level of NRF 2 normalized to GAPDH (n = 3); J. RNA level of HO 1 normalized to GAPDH (n = 3). * P < 0.05, ** P < 0.01, *** P < 0.001.

We finally explored the specific mechanisms by which S1R stimulation could affect the morphological and functional variations mentioned above. Western blotting was used to reveal the expression level of S1R, aiming to determine its predominant role. MCT dramatically decreased S1R expression by approximately 87.5% compared with baseline in the RV (Figure 5a,5b), which was partly restored by fluvoxamine administration in the P + F group (Figure 5a,5b).

NADPH oxidases (NOX) are one of the most common sources of ROS in the heart. NOX 2 and NOX 4 expression was significantly upregulated in the RV (Figure 5a,5c) and (Figure 5a,5d) by PAH. The difference from the above results was that fluvoxamine did not affect either NOX 2 expression (Figure 5a,5c) or NOX 4 expression (Figure 5a,5d) of the PAH rats in the P + F group. The above results indicated that NOX expression did not participate in the process of activating S1R to alleviate oxidative stress in PAH. Advanced ROS concentration impaired the expression of NRF 2. As expected, NRF 2 expression in the RV was strongly downregulated by PAH, as exhibited in the PAH group compared with the CTL group (Figure 5a,5e). In addition, the RNA level of NRF 2 displayed the same variation in the two groups (Figure 5h). The protein and RNA levels of NRF 2 were measurably rescued by S1R activation in the P + F group (Figure 5a, 5e, 5h) Similarly, HO 1 expression was downgraded dramatically in the PAH group (Figure 5a,5f,5i) but partly recovered in response to fluvoxamine in the P + F group (Figure 5a,5f,5i) (Figure 5).

Compared with the CTL group, fibrosis in the PAH group was further proven by the upgraded level of collagen I in the RV (Figure 5a,5g). In contrast to tissues in the PAH group, collagen I expression was dramatically decreased by fluvoxamine (Figure 5a,5g), reinforcing the reduction in fiber deposition.

## Discussion

Pulmonary arterial hypertension (PAH) is a syn- drome characterized by progressive remodeling of pulmonary arterioles, luminal stenosis, and endothelial dysfunction, inducing RV remodeling and heart failure. Therefore, it is necessary to reduce RV dysfunction to avoid severe cardiovascular consequences. To date, the underlying mechanism of PAH has not been fully elucidated. The present results showed that (1) Sigma 1 receptor (S1R) was expressed in the RV and was downregulated in PAH rats; (2) pharmacologically activated S1R alleviated right ventricular dysfunction and structural remodeling induced by pulmonary hypertension; (3) S1R activation did not reduce pulmonary vessel remodeling in rats with PAH; and (4) S1R exerted its protective effects partly through oxidative stress mediated by the NRF 2/HO 1 pathway.

Sigma 1 receptor (S1R) was initially found to be expressed in the central nervous system, dysregulated in multiple neurological and psychotic diseases, and targeted to treat neuropsychiatric disorders. [14,34–36] An increasing number of studies have demonstrated that S1R is expressed in the heart and lung. [14,24]. In addition, studies in recent years have stressed the infrastructural role of S1R in the left ventricle. Sig-1 R KO mice showed mitochondrial dysfunction and abnormal cardiac remodeling and eventually systolic dysfunction, stressing the fundamental need for maintaining systolic function.[20] Furthermore, fluvoxamine remarkably rescued heart failure and cardiac dysfunction in transverse aortic constriction animal models by activating S1R.[14], ^15^ S1R was also documented to be highly expressed in the right ventricle.[14] However, to the best of our knowledge, little is known about the role of S1R in the RV, especially in RV dysfunction.

Oxidative stress was found to increase in the lungs of both PAH patients and the experimental rodent PAH model and contribute to PAH development. [4–6] Oxidative stress has also been suggested to play an essential role in pulmonary vascular remodeling. Pharmacological targeting of oxidative stress for PAH treatment has been explored, and drugs reducing ROS production can improve PAH structural remodeling and halt PAH progression. [5,37,38] The activity of NRF 2 was increased in the oxidative stress response to mitigate the damage of excessive ROS. [10,11] Several NRF 2 activators, such as dimethyl fumarate and bardoxolone methyl, have been shown to alleviate pulmonary vascular remodeling, inflammation, and fibrosis and prevent the progression of pulmonary hypertension. [12,13,39] S1R was shown to maintain the balance of ROS both in the physiological state of the lung and the liver[24] and in pathological conditions such as retinal degenerative disease[25], lipopolysaccharide-induced astrocyte damage,[40] and amyotrophic lateral sclerosis[41]. Furthermore, in retinal degenerative disease and astrocyte damage, the protective effect of S1R was mediated by restoring maladjusted NRF 2 activation. [25,40] Similarly, in our PAH model, S1R also decreased the elevated ROS level in the RV, which was not mediated by altering the expression level of NOX 2 and NOX 4, two important resources of ROS, but was mediated by increasing the NRF 2 level. However, in the current study, S1R activation did not improve pulmonary vascular remodeling due to because there was no S1R expression in the pulmonary arterioles.

RV fibrosis impairs the conduction of electrical activity between cardiomyocytes, resulting in dyssynchrony of right ventricular cardiomyocytes and ultimately a decline in RV systolic function, similar to fibrosis in the LV. S1R activation mitigated fibrosis in the RV of PAH animals, as shown by Masson’s staining, and collagen I deposition was reduced in the P + F group. A similar reduction in fibrosis of the LV was observed in our previous study and other studies in several left heart disease animal models. [20,42]

Hypertrophy of the RV has been observed in several experimental studies of PAH. [26,29,43] Similar to the antihypertrophic effect in the pressure-overload-induced heart failure model [14,15], there was less hypertrophy in the fluvoxamine-treated PAH rats. The reduced hypertrophy in the P + F group rats may be due to the improvement of RV function, which leads to the degradation of the compensatory hypertrophy program that initially compensates for the high RV afterload. In addition, the decreased oxidative stress also contributed to the antihypertrophic effect of S1R activation because oxidative stress is related to the degree of hypertrophy.[4]

RV function is a key determinant of outcome and prognosis in patients with PAH, not the estimated systolic resting systolic pulmonary arterial pressure.[1] RV dysfunction in PAH rats was characterized by deceased RV EDP reduced TAPSE, and elevated NT-pro-BNP concentrations. S1R activation by fluvoxamine improves RV function, as evidenced by the relief of indicators of right ventricular function in the above responses, which was similar to the preferable protective effect of fluvoxamine on left ventricular function. [14,15] The Constant pulmonary arteriole remodeling and consistent pulmonary arterial pressure did not relieve the afterload of the RV, suggesting that S1R mainly acted through direct action.[29] In addition, a reduction in oxidative stress injury and improvement in downstream fibrosis and hypertrophy of the RV further stressed the protective effect of S1R on RV systolic function. [12,13] Furthermore, impaired glucose metabolism is another characteristic of RV dysfunction secondary to PAH. ^26^ S1R activation elevated the ATP level of hypertrophic cardiomyocytes, so repairing metabolic disturbances may contribute to the preferable effect of S1R.[44]

In conclusion, our study demonstrated that S1R receptor expression in RV was downregulated in PAH, and continuous activation of the receptor could alleviate the the structural changes and dysfunction of the right ventricle without affecting pulmonary artery pressure and remodeling. Furthermore, the protective effect of S1R was mainly mediated by the antioxidant stress NRF 2/HO 1 pathway. Therefore, our study provides a potential therapeutic target for the clinical treatment of right ventricular dysfunction.

## Limitations

We acknowledge the following deficiencies. First, MCT animal models cannot completely replicate the variations in the lungs and pulmonary vessels in patients with PAH; however, they are very effective for exploring the in-depth mechanism of PAH and validating new target therapeutic approaches. Second, we did not proceed with a genetic intervention of S1R, but fluvoxamine has been proven to have the highest known affinity for S1R and is widely used to study S1R in numerous diseases.[14] Further studies are needed to explore the role of S1R in RV and its underlying pathological mechanism.

## Conclusions

Our study demonstrated that chronic S1R activation decreases fibrosis, hypertrophy of the RV, and improves RV function without affecting pulmonary artery pressure.

## Supplementary Material

Supplemental Material

## Data Availability

Data will be made available by the corresponding author upon request.
