# Supplementary material for: Chronic Sigma 1 receptor activation alleviates right ventricular dysfunction secondary to pulmonary arterial hypertension
Source: Bioengineered. 2022 Apr 27;13(4):10843–56. doi: 10.1080/21655979.2022.2065953 (PMC9208487; doi:10.1080/21655979.2022.2065953)
Supplement: Supplemental Material [file KBIE_A_2065953_SM8659.zip › supplementary/data.docx]

|  | CTL | Flu | P value vs.CTL | PAH | P value vs.CTL | P+F | P value vs.PAH |
| --- | --- | --- | --- | --- | --- | --- | --- |
| RV EDP (mmHg) | 4.34±1.29 | 2.45±1.25 | 0.32 | 9.60±2.09 | ＜0.001 | 5.90±1.94 | 0.02 |
| RV ESP (mmHg) | 22.82±2.31 | 24.12±8.20 | 0.99 | 62.71±22.93 | 0.009 | 54.89±23.06 | 0.88 |
| TAPSE (mm) | 3.33±0.09 | 3.23±0.07 | 0.3 | 2.17±0.22 | ＜0.001 | 2.82±0.20 | ＜0.001 |
| FAC(%) | 50.53±2.05 | 48.68±5.62 | 0.53 | 30.24±6.75 | ＜0.001 | 41.35±4.17 | ＜0.001 |
| LVEF(%) | 81.98±2.64 | 80.74±4.52 | 0.58 | 82.06±3.57 | 0.97 | 80.60±4.23 | 51 |
| NT-pro BNP(pg/ml) | 0.51±0.10 | 0.53±0.12 | 0.99 | 1.63±0.17 | ＜0.001 | 0.80±0.23 | ＜0.001 |
| mPAP(mmHg) | 17.68±3.94 | 19.01±2.94 | 0.99 | 43.22±11.78 | 0.002 | 43.29±13.25 | ＞0.99 |
| WA%(%) | 35.66±6.22 | 36.52±6.21 | 0.53 | 86.39±4.06 | ＜0.001 | 84.55±4.24 | 0.19 |
| WT%(%) | 21.26±2.99 | 21.03±6.34 | 0.89 | 64.98±7.15 | ＜0.001 | 64.83±7.63 | 0.92 |
| Fibrosis in perivascular space of RV(%) | 2.38±0.63 | 2.28±0.05 | 0.87 | 11.04±0.23 | ＜0.001 | 3.18±0.51 | ＜0.001 |
| Fibrosis in interstitial space of RV(%) | 1.08±0.05 | 1.13±0.17 | 0.93 | 7.23±1.26 | ＜0.001 | 2.06±0.19 | ＜0.001 |
| Mean cross-sectional area(μm^2^) | 244.36±10.14 | 246.13±9.68 | 0.95 | 560.25±18.84 | ＜0.001 | 416.32±28.38 | ＜0.001 |
| RV/(LV+S) | 0.32±0.02 | 0.34±0.02 | 0.98 | 0.76±0.04 | ＜0.001 | 0.58±0.01 | ＜0.001 |
| OD value | 49.31±1.34 | 57.09±5.63 | 0.1 | 74.43±3.51 | ＜0.001 | 52.70±4.54 | ＜0.001 |
| MDA (nmol/ml) | 9.09±2.90 | 8.83±3.16 | 0.99 | 27.30±6.70 | ＜0.001 | 15.77±2.65 | ＜0.001 |
| SOD(U/ml) | 326.27±58.61 | 329.07±57.11 | 0.99 | 66.34±19.04 | ＜0.001 | 210.75±30.16 | ＜0.001 |
| S1R/GAPDH | 0.72±0.03 | 0.73±0.04 | 0.81 | 0.09±0.01 | ＜0.001 | 0.35±0.01 | ＜0.001 |
| NOX 2/GAPDH | 0.13±0.03 | 0.14±0.05 | 0.96 | 0.57±0.08 | 0.004 | 0.51±0.12 | 0.61 |
| NOX 4/GAPDH | 0.16±0.04 | 0.15±0.05 | 0.94 | 0.79±0.10 | 0.005 | 0.69±0.19 | 0.56 |
| NRF 2/GAPDH | 0.60±0.09 | 0.61±0.07 | 0.96 | 0.09±0.02 | ＜0.001 | 0.31±0.04 | 0.03 |
| HO 1/GAPDH | 0.64±0.03 | 0.63±0.03 | 0.81 | 0.086±0.02 | ＜0.001 | 0.22±0.02 | 0.005 |
| Collagen I/GAPDH | 0.14±0.02 | 0.14±0.03 | 0.95 | 0.53±0.07 | ＜0.001 | 0.31±0.02 | 0.006 |
| Fold change in expression of NRF 2 | 0.99±0.018 | 0.91±0.02 | 0.002 | 0.44±0.03 | ＜0.001 | 0.72±0.02 | ＜0.001 |
| Fold change in expression of HO 1 | 1.08±0.11 | 1.00±0.10 | 0.22 | 0.46±0.03 | ＜0.001 | 0.69±0.03 | 0.006 |
