# Supplementary figures and images for: Chronic Sigma 1 receptor activation alleviates right ventricular dysfunction secondary to pulmonary arterial hypertension
Source: Bioengineered. 2022 Apr 27;13(4):10843–56. doi: 10.1080/21655979.2022.2065953 (PMC9208487; doi:10.1080/21655979.2022.2065953)

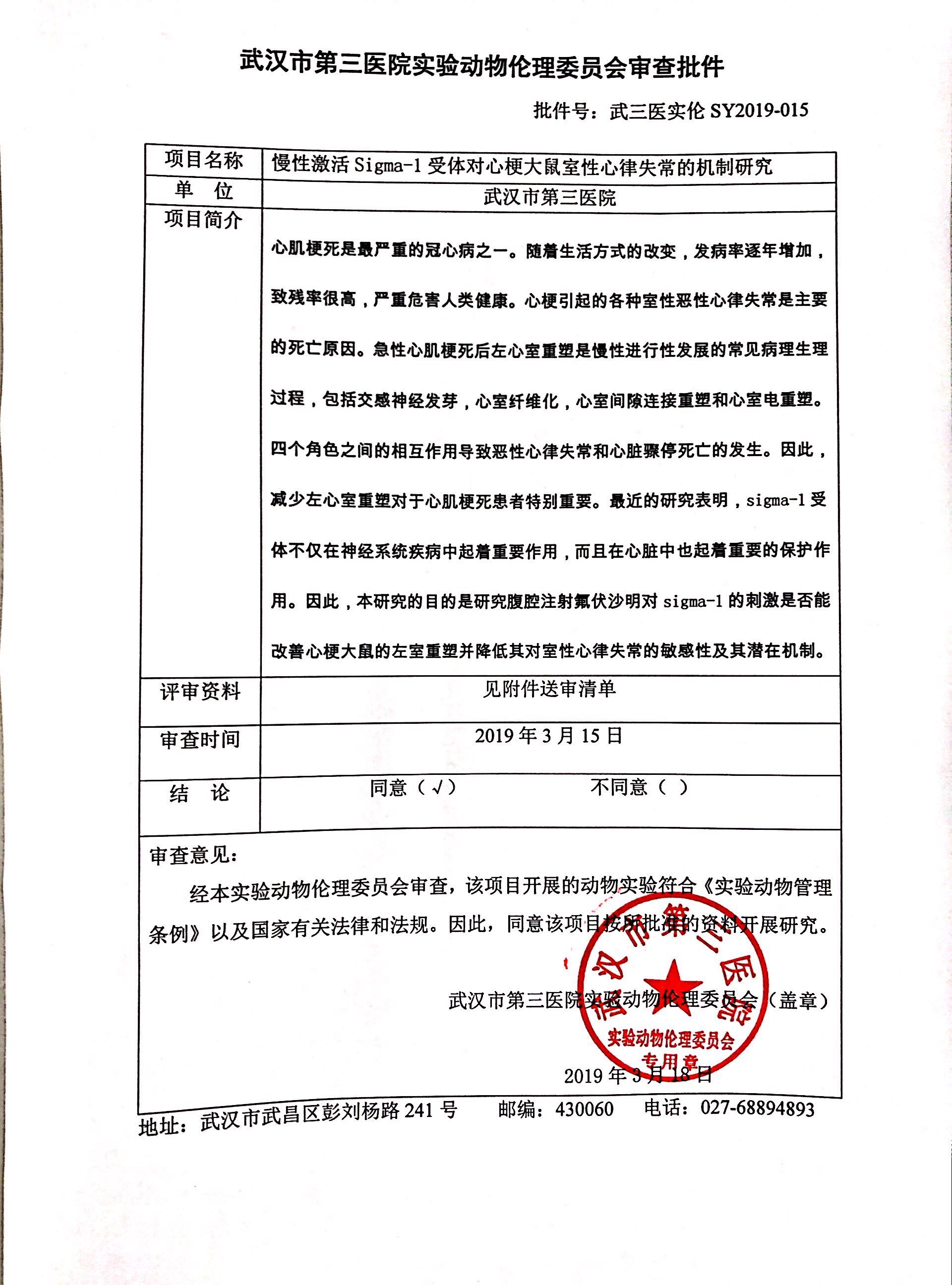

Supplement: Supplemental Material [file KBIE_A_2065953_SM8659.zip › supplementary/ethical approvement.jpg]

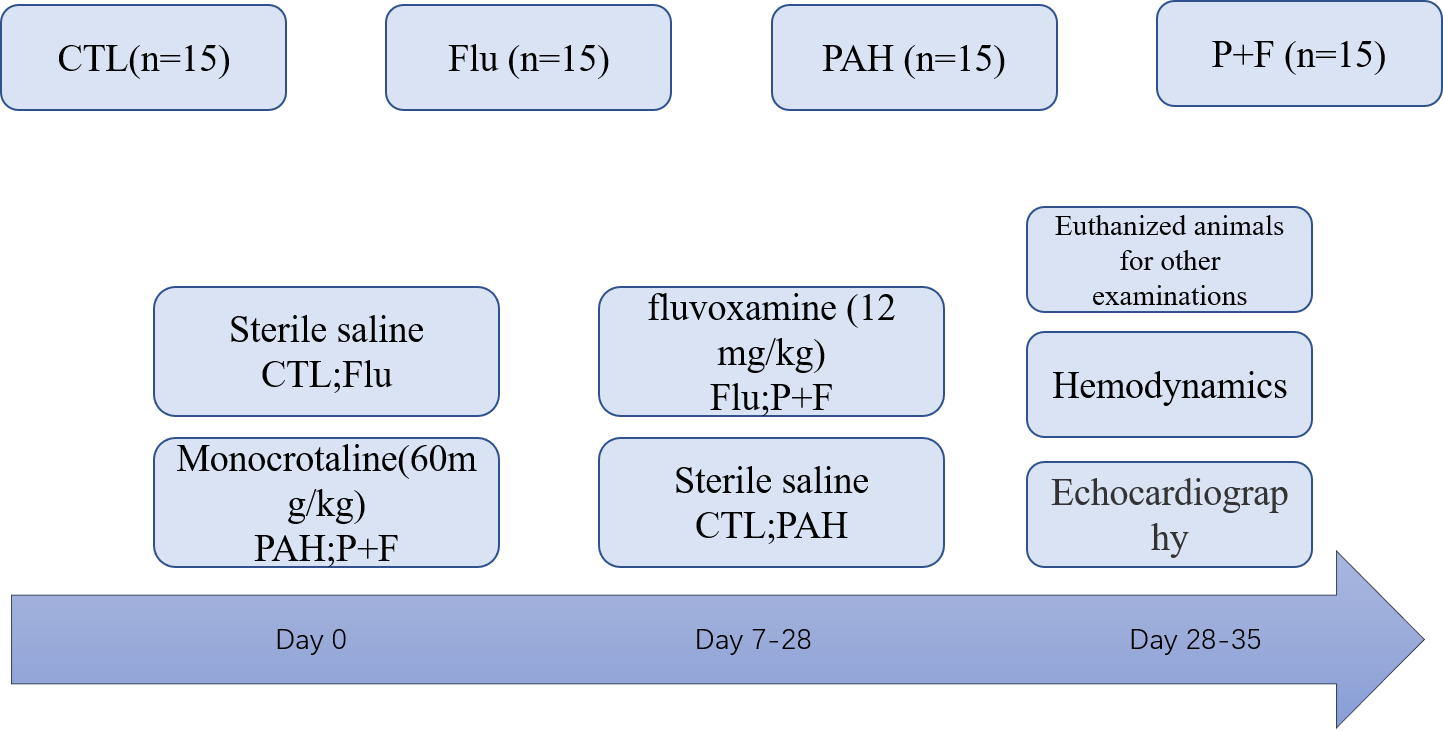

Supplement: Supplemental Material [file KBIE_A_2065953_SM8659.zip › supplementary/flow sheet abstract.tif]
